# Professionals’ views on working in the field of domestic violence and abuse during the first wave of COVID-19: a qualitative study in the Netherlands

**DOI:** 10.1186/s12913-021-06674-z

**Published:** 2021-06-30

**Authors:** Nicole E. van Gelder, Ditte L. van Haalen, Kyra Ekker, Suzanne A. Ligthart, Sabine Oertelt-Prigione

**Affiliations:** 1 Gender Unit, Department of Primary and Transmural Care, Radboud University Medical Center, Postbus 9101, Nijmegen, Netherlands; 2 Nijmegen, Netherlands

**Keywords:** COVID-19, Coronavirus, Pandemic, Domestic violence and abuse, Child abuse, Intimate partner violence and abuse, Working conditions, Occupational health, eHealth, Online

## Abstract

**Background:**

The COVID-19 pandemic and lockdown evoked great worries among professionals in the field of domestic violence and abuse (DVA) as they expected a rise of the phenomenon. While many countries reported increased DVA, the Netherlands did not. To understand this discrepancy and the overall impact of the lockdown on DVA support services, we interviewed DVA professionals about their experiences with DVA during the rise of COVID-19, the impact of the lockdown on clients and working conditions, and views on eHealth and online tools.

**Methods:**

Semi-structured interviews were conducted among 16 DVA professionals with various specializations. This data was analyzed using open thematic coding and content analysis.

**Results:**

Most professionals did not see an increase in DVA reports but they did notice more severe violence. They experienced less opportunities to detect DVA and worried about their clients’ wellbeing and the quality of (online) care. Furthermore, their working conditions rapidly changed, with working from home and online, and they expressed frustration, insecurity and loneliness. Professionals feel eHealth and online tools are not always suitable but they do see them as an opportunity to increase reach and maintain services when physical contact is not possible.

**Conclusion:**

This study suggests DVA was probably under-detected during the lockdown rather than not having increased. The Dutch system heavily relies on professionals to detect and report DVA, suggesting a need for critical evaluation of the accessibility of professional help. Professionals experienced significant challenges and should themselves be supported psychologically and in their changed work practices to maintain their ability to aid survivors.

**Supplementary Information:**

The online version contains supplementary material available at 10.1186/s12913-021-06674-z.

## Background

On 27 February 2020, the Netherlands officially detected the first Dutch patient infected with the novel coronavirus (SARS-CoV-2) [1]. The virus causes COVID-19 disease and was first reported in Wuhan (China) in December 2019 [2]. In the beginning of March 2020, the Netherlands implemented the first measures to combat the spread of COVID-19 [3] culminating in the so-called ‘intelligent lockdown’ on 23 March. This approach entailed measures such as working from home with the exception of essential workers, closure of restaurants, clubs, hairdressers, gyms, and schools, limitations in traveling and meeting other people [4]. Supermarkets and pharmacies remained open at all times, essential and emergency services continued to be available, people could still visit the general practitioner (GP), they were allowed to meet a small number of people (limiting contacts was strongly advised), and they could go outside to exercise. In May and June, the rules were relaxed [5, 6] and since September increasingly stricter rules are in effect again [7]. Overall, the Dutch lockdown was more lenient than the lockdowns in France, Italy and Spain but comparable to Germany and Belgium [8–10].

With the implementation of lockdowns across the world, worries emerged about a rise in domestic violence and abuse (DVA) cases [11, 12]. These worries were based on earlier knowledge about the association of pandemics, disasters, lockdowns, isolation measures, economic stress (job loss, financial problems) with increased DVA, including intimate partner violence and abuse (IPVA) and child abuse [13]. In line with this prediction, many countries, including Italy, Germany, the United Kingdom (UK), Argentina, the United States (US), Singapore, Tunisia, and Australia reported increases in DVA [14–18]. In the Netherlands however, current official reports of the police and *Veilig Thuis* (VT; translation: Safe At Home; national DVA organization) did not register an increase of DVA [19–21]. Some other (DVA) organizations such as *Fier* (translation: Proud), *Sterk Huis* (translation: Strong Home) and the *Kindertelefoon* (translation: Childline) did report a rise in child abuse and sexual violence since the first lockdown [20, 22, 23]. At the time of the lockdown, the Dutch government launched a national campaign to raise awareness on DVA and report options. Pharmacies were instructed to call for help if customers used the codeword “mask 19” [21, 24] and VT and several local DVA organizations started providing chat options [25, 26]. The chat option of VT was frequently used [27].

DVA professionals in the Netherlands expressed concerns about not being able to have direct contact and having less options to notice signs of DVA. Measures such as school closures, reduced GP visits, reduced home visits by social workers and police all have the potential to decrease the opportunity for detection of situations of abuse. In fact, pre-pandemic data demonstrate that 61% of all DVA reports to VT come from the police [28]. Most studies on DVA during the pandemic focus on victims / survivors (from here on referred to as survivors), yet professionals working with survivors were significantly affected by the pandemic as well. Professionals had to adapt to changed working conditions as a result of the COVID-19 measures, for example working from home and using online tools [19, 29–31]. Little is known about the impact of the pandemic on DVA professionals. We set up the current study to investigate the experiences of professionals with regard to DVA, including the impact on clients, working conditions, and eHealth / online tools use in the context of the COVID-19 pandemic.

## Methods

### Study design and data acquisition

The current project was designed to answer the following questions:

- *What are professionals’ experiences with and views on domestic violence, including the changes in incidence during the rise of the COVID-19 pandemic?*
- *How has the professionals’ work changed during the COVID-19 pandemic, and how do they deal with these changes?*
- *What are the professionals’ experiences with and views on using eHealth / online tools during the COVID-19 pandemic in the context of domestic violence?*

This qualitative study consists of semi-structured interviews with 16 professionals and experts in the field of DVA or who encounter DVA in working with patients / clients, for example professionals from (mental) health care, DVA organizations, and the police. One researcher (KE; currently doing a medical residency, with previous experience of processing interview data as a research assistant) conducted all the interviews after being trained by a researcher with previous experience in interviewing (NvG) and was supervised during three interviews. A flexible interview guide (see Additional file 1) was used containing questions on the professionals’ experiences in the COVID-19 pandemic regarding DVA, working conditions, and the use of online tools / eHealth, such as: “Have you seen an increase in DVA or IPVA in the pandemic?”, “Has your working day changed since the pandemic? If so, can you explain how?”, and “Do you think online tools / eHealth is a good (supplemental) option during the pandemic? And why?”. Before the start of the interview the participant received an information letter and signed an informed consent form. Interviews were conducted in Dutch and took place online with a duration between 30 and 45 min. Each interview was recorded (audio only) and typed out *ad verbatim*. All data was gathered in the period between 20 August 2020 and 8 October 2020. The Dutch quotes were translated into English in this manuscript. The study obtained approval from the local ethics committee (*Commissie Mensgebonden Onderzoek regio Arnhem-Nijmegen*) on 6 April 2020 and is conducted in accordance with the Declaration of Helsinki.

### Recruitment and study population

In the context of the development of an eHealth intervention (SAFE) [32] a record was kept of professionals contacting the project team for professional networking, updates and general information. Several interviewed individuals were recruited from this database. Furthermore, we reached out to professionals with a call on the internet platform LinkedIn. We recruited 16 participants to underwrite code and meaning saturation [33]. We interviewed 14 women and 2 men from various organizations, occupations and regions in the Netherlands, for example VT (Dutch national DVA organization), professionals with specific tasks regarding DVA and child abuse (in Dutch: *aandachtsfunctionaris huiselijk geweld en kindermishandeling*) at a hospital, and Sexual Assault Center (Table 1). The regions of work varied from nationally to specific provinces or regions, covering seven of the 12 Dutch provinces.

**Table 1:** Description of the study population (*N* = 16)

Participant	Sex	Age group	Occupation
101	Female	31–40	Psychologist
102	Female	41–50	Social worker at a DVA organization
103	Female	51–60	Children’s coach
104	Female	41–50	Founder of a foundation for survivors of DVA
105	Female	31–40	Working at a police department as a specialist on child abuse
106	Female	20–30	Working at the Ministry of Health, Welfare and Sport
107	Female	20–30	Policy advisor on DVA and child abuse at a municipality
108	Male	31–40	Trainer on the subject of honor based violence
109	Male	31–40	Project leader for online care at a DVA organization
110	Female	41–50	Project leader for a local initiative on DVA and child abuse
111	Female	51–60	Physician and systemic therapist, specialized in DVA and child abuse
112	Female	51–60	Working at a hospital as a specialist on DVA and child abuse
113	Female	51–60	General practice mental health worker (in Dutch: *POH GGZ*^*a*^)
114	Female	31–40	Trainer on the subject of DVA and child abuse
115	Female	41–50	Social worker
116	Female	31–40	Working at the Sexual Assault Center

^a^Praktijkondersteuner Geestelijke Gezondheidszorg

### Analysis

All interviews were coded with open thematic coding and analyzed through deductive content analysis [34, 35]. The researchers (KE and NvG) and a research assistant coded the interviews independently using Atlas.ti version 8.4 [36], resulting in each interview being coded two or three times. All personal identifiers were removed to prevent recognizability of individual participants from the illustrative quotes. Four consultation rounds were held for comparing the codes and quotations and reaching consensus regarding the coding frame. After finishing the coding process, the final codebook was used to read all interviews again and check for completeness of the coding process. The interview guide contained three main themes that provided the pre-determined analysis framework for the coding process: domestic violence, working conditions, and the use of eHealth / online tools. The subthemes, such as ‘types of violence’ and ‘clients and groups at risk’, were determined during the coding and analysis process.

## Results

In total, 16 professionals and experts were interviewed in the context of the COVID-19 pandemic, about the following three (predefined) main themes: a) Domestic violence during the COVID-19 pandemic, b) Working conditions during the COVID-19 pandemic, and c) The use of online tools / eHealth during the COVID-19 pandemic. Table 1 displays the various types of professionals we interviewed ranging from policy, to (mental) healthcare professionals, to DVA trainers. Some professionals work directly and explicitly with survivors of DVA while others work more indirectly in the field of DVA or have no explicit focus on DVA but do encounter it. One professional focused specifically on honor based violence and another professional had an explicit focus on sexual violence.

### Domestic violence and abuse during the COVID-19 pandemic

Overall, professionals said they did not see a rise in DVA during the first wave of the COVID-19 crisis in the Netherlands. Four professionals mentioned that the numbers from *Veilig Thuis* (VT) do not show increased DVA either. In fact, shortly after the first lockdown was announced, professionals at various organizations saw a decline in reports for a short amount of time. Nine professionals feared that these numbers might not be fully representative of the actual situation. They talked about signs that led them to believe that DVA might be worse during this pandemic than the official numbers currently show, caused by people experiencing more stress, loneliness and hopelessness, and worries about COVID-19.

*115: “At some point I noticed that families which already had tension had trouble keeping their head above water. You have to stay home, you have nowhere to go, they started to get a short temper. We definitely noticed that, but the real increase that has drawn nation-wide attention, and we regularly talked about that with VT, we did not have that in our region.”*

Some professionals were especially worried about children being in a vulnerable position during the school closure. Two professionals noticed that people start using more alcohol or drugs or using it earlier on in the day. They described more excessive violence and crisis situations compared to before the first lockdown. One professional questioned whether the violence itself is becoming worse or if this is due to delayed early intervention opportunities due to COVID-19 measures.

*105: “But there were a few situations in which I thought, ‘Jesus…’. I wonder if the injury would have been this severe in a different situation, not during corona, or if it wouldn’t have come this far. (…) People are somehow reluctant to call.”*

*108: “These incidents have clearly become more severe, because it was impossible to intervene in an earlier stage. So, we couldn’t identify it in a timely manner to defuse the situation in that moment.”*

Furthermore, professionals at local DVA organizations saw a surge in the use of online chats. One professional said that chat conversations at his organization increased by almost 40% since the start of the lockdown. This mainly applied to sexual violence and child abuse, reports of IPVA seemed to have declined. One professional stressed the importance of these chats, especially for survivors who are stuck at home with the perpetrator and have no other means of asking for help. Two professionals at different organizations saw an increase in the total number of phone calls as well, for example from bystanders asking for advice or survivors calling for help, although some people call multiple times.

*109: “What we see on the chat anyway is that 35% on this comes from survivors themselves. Meaning people who got in the situation. And they, yeah they had little opportunities for refuge, except for, one of these opportunities is thus an anonymous possibility to chat.”*

Some professionals feared that there actually is an increase in DVA but that a substantial part of this remains hidden.

*101: “I can hardly imagine that that [an increase in DVA] is not happening, but the question is whether we would see it, identify it and if we are able to anticipate on it. And we have not seen it, but that doesn’t mean it is not happening.”*

The professionals proposed three explanations for why a possible increase might not have been detected:

- There was a lack of interaction due to COVID-19 measures on several levels. For example, most reports at VT come from the police, who only entered houses in serious crisis situations, resulting in less opportunities to recognize signs of DVA and less reports to VT. Also, other professionals (such as social workers) stopped doing home visits and used video calls or other online means instead, making it harder to notice risky situations and signs of DVA, which could have led to an underreporting of DVA. Third, teachers play an important role in noticing and reporting signs of child abuse. When schools closed and proceeded with online education, teachers had less insight in their pupils’ wellbeing and sometimes lost all contact with certain pupils, possibly resulting in less DVA reports.
  *102: “Yes, those first three weeks there was a decrease [in DVA reports] and after that it increased again. And slowly it is getting back to the way it was. Especially because of course the majority of reports come from the police. And they’re back to work as usual.”*
  *105: “At the beginning you’re reluctant to go inside. When they [the police] have to go inside, they will just go inside. But when you do not absolutely have to and you are able to solve the issue in a different way, we rather did that. While I think that we, as police, should always go inside. Even if we have a very calm and good conversation, we should go inside. (…) That is the most important thing to do, to really observe the situation.”*
- The second explanation is a lack of opportunity to ask for support: it could have been harder to ask for help when families were confined at home together during the lockdown.
  *109: “Well, it could be that, because they’re in each other’s pockets more and more, it becomes harder to find help. That could be a possible explanation.”*
- Last, it is possible that there has been no actual increase: many families found themselves already in stressful, isolated situations and COVID-19 would just be another stress factor, with relatively little impact. Second, if there was an actual increase in DVA, professionals should have noticed this when they returned to their normal way of working during the relaxation of the rules.
  *111: “No and when I look at the families I work with, domestic violence has been an issue for years already and corona is not a factor. Of course, this adds to an explosion, but there are also other moments in which it explodes. (…) Perhaps we all have been more alert on this during corona. It has maybe been given a lot of publicity, deservedly. (…) Corona is also a stress factor for people causing it to explode, but it was already there.”*

In relation to the aforementioned reasons, one professional stated that the system relies on professionals too much in detecting DVA. A few professionals agreed that survivors often do not ask for help themselves. Survivors are afraid to call VT or the police, worried about the consequences, and instead do not ask for help or contact other organizations.

*107: “But it tells me something about how we set up the system, namely that we are so dependent on professionals to report domestic violence. And when those professionals cannot visit people in their homes, who will do it then? So in that regard, I wonder if the lower reports at VT represent the reality. (…) We now see that very little people who are survivors or bystanders themselves ask for help. ”*

Some professionals still expected a rise in DVA if this crisis continues for much longer, due to economic difficulties and a higher level of unemployment leading to more stress. They feel tension builds over time and at some point, is likely to escalate at home. One professional was particularly worried about elderly abuse because family caregivers have been under increased pressure during the pandemic. Moreover, ending the lockdown will lead to a partial return to normal life, but since certain restrictions will remain in place, finding a new balance could be stressful and increase the risk of DVA.

*112: “You have to justify why you’re doing the one thing and why you’re not doing the other thing. Why do or do not I follow the rules, what do I think about the restrictions. There is much more conversation going on in which people can disagree, also among each other. (…) Yeah my expectation was, is indeed that especially in the period of lockdown it [DVA] would be less and in the period we had just now, in which life partly starts getting back to normal, that that would give much more tension.”*

### Working conditions during the COVID-19 pandemic

Working conditions changed for all professionals in this study due to COVID-19 measures. Many of them had to start (mainly) working from home and / or working online. Not all organizations were prepared for this and some problems or adaptations to the new work requirements were discussed by the professionals. Several issues were identified, for example use of different systems for online calls, internet quality, rapid changes of plans, and how to maintain a connection with (potential) survivors of DVA.

*101: “At a certain point we did online admissions but that’s far from ideal because you’re missing a lot information. The connection wasn’t always working properly. Our network wasn’t prepared for people working online at all so it was overloaded.”*

*102: “Working online was hard because of course it had taken us all by surprise. (…) Each organization uses a different system and we are very strict when it comes to privacy, thus, we could only videocall clients via protected systems. And well, the quality [online and via telephone] left much to be desired which caused a lot of frustrations for the people involved. It was a quest for professionals, abiding privacy and safety rules but also wanting contact with clients with minimal frustrations.”*

*106: “At the peak of the corona crisis we communicated a lot with societal parties about the situation and what extra steps to take. That has led us to accelerate some processes and to start up some processes that wouldn’t have happened otherwise.”*

Appreciation of online contact by professionals and clients varied. Three professionals stated it worked well while many others expressed reservations about its effectiveness compared to face-to-face contact. They said clients had varied experiences as well. Professionals thought many of them would prefer face-to-face contact, but others might like the flexibility and reduced travel time that online contact provides.

*111: “What I find positive is videocalls for network advisory boards, so talking with the involved organizations and the family at the same time. I still use videocalls for this and that actually works very well. It is more organized and it is easier to make agreements on safety because people wait for their turn to talk.”*

*113: “(…) people can choose what they want. For example, I have a videocall planned with a truck driver who cannot come during the day so we do it this way. Flexibility is nice, although not everyone likes it. We do notice it works well and (…) I can see the look on your face and your body language perfectly fine. But often clients also like the physical proximity, their opinions vary.”*

Contact reduction with clients is perceived as an obstacle by many professionals. They felt less involved and said that this change was hard for both clients and professionals. In cases of online or phone appointments, sometimes the treatment context or the situation at home led to challenges. Furthermore, not all appointments and treatments could continue, leading to less contact with clients and less options to notice signs of DVA.

*105: “What I think is that when you work from a distance you lose involvement. Not because people suddenly are not involved but you have so little interaction. If you’re with a family you feel the energy, it is very different from a phone call. We do not solely exist of hearing, we have all these other senses. I think you miss incredibly much.”*

*101: “The clients said it was very hard, all hands on deck. And what I notice is that being at home a lot hinders treatment. I said ‘I’d rather have no children present during treatment’ but then a child enters the room or the children are arguing with each other downstairs. So it was hard for parents to make time for their treatment.”*

While the workload had not necessarily increased for all professionals, they experienced challenges at multiple levels that are intertwined: a) emotional and b) practical.

- On the emotional level, many professionals experienced increased stress and worrying. They were concerned about their clients wellbeing. Two professionals experienced loneliness due to their new work environment: working from home and therefore having less contact with their colleagues.

*107: “When corona hit, we quickly started worrying ‘if we’re all in quarantine, what will happen in the families where it [DVA] is or could be present?’. It was an enormous challenge because we had to make new agreements with social workers, what could VT do, what about home visits? It was actually a complete organizational reset, a lot of extra work.”*

*102: “The workload has declined now we can do telephone shifts at the office again. It really was a big psychological burden to do that from home. It is nice to discuss some things with a colleague, it reduces stress and then you can go ahead with the next call. Doing this from home, you just go on with taking calls, you do not call a colleague.”*

b) On the practical level, many professionals had to get used to a new way of working: working online. This also meant less face to face contact with clients and colleagues which added to feelings of loneliness for some professionals. Furthermore, a few professionals expressed challenges in maintaining a healthy work-life balance.

*101: “(…) I found talking to a screen all day very exhausting but not necessarily the amount of work. It cost more time in the beginning to get all the technical things in order, that was a greater burden. At first I worried about the quality of treatments going down, I was skeptical about online treatment. I thought ‘we’re not at all ready for this yet, how am I going to manage this?’. But I’m confident I was able to provide sufficient care, although it wasn’t ideal and some treatments temporarily stopped.”*

*113: “When you work from home a lot, my husband’s working a lot from home now, I find it becomes a bit boring, a bit lonely. Even though I cannot say I spend less time now on work contacts. I do exactly the same as I would with someone sitting opposite me. I do not get people at all who say that they have more free time. I find it very intensive.”*

Four professionals said they sometimes had to stop certain types of care or that admissions stopped. Some professionals weren’t always able to perform certain tasks that are part of their normal work situation, for example providing workshops or specific treatments. Partially because of this, two professionals said they were unable to provide sufficient care and support.

*101: “Some treatments were on-hold because we were careful, for example trauma treatments. If you’re in the same room and someone gets overwhelmed by emotions you anticipate and help regulate. But if someone’s at home and you lose the connection while doing an exercise… There are many studies that prove that online treatments can actually work quite well but of course for us it was a sudden switch.”*

Three professionals talked about measures that their organizations took on location, for example disinfection, distancing, and one-way traffic in hallways. One of them also talked about how they sometimes had to deal with clients who disagree with the organization’s decisions regarding COVID-19 measures.

*101: “We had rules like one-way traffic, disinfection, 1,5 meter distance etcetera. A significant part of my clients think it is nonsense but I think we have to be careful. Some people oppose the rules. For example, yesterday a client needed to use the toilet but because of the one-way traffic rule he had to make a detour to get there. That man really caused trouble, he thought it was rubbish. But I have colleagues who are vulnerable due to chronic illnesses so it is important that everyone follows the rules.”*

Many professionals said that their working conditions are (somewhat) returning back to normal when we interviewed them at the end of August till the beginning of October. They started working face-to-face with people again.

*114: “I give a training face-to-face again because it fits within the allowed group size and people have the room available. But I’ve primarily worked online.”*

### The use of online tools / eHealth during the COVID-19 pandemic

Most professionals were familiar with eHealth, at least to some degree, and five of them already used eHealth modules in their trainings or treatments, sometimes directly in the context of DVA. Many of them now used online tools for online contact, such as video calls or chats, and some organizations designed these tools or eHealth modules specifically to provide support during the pandemic.

*101: “We already used eHealth for psycho-education, monitoring, teaching skills, pre-treatment, and as a means of communication. The organization wants to expand this, I think it is also a prerequisite from the health insurers. Since corona this has increased, we also use it for videocalls. Besides that, the organization developed three modules specifically for corona time, on coping skills or to create structure in daily life. But I think we will increase the use anyway. I notice that people find it easier now while before there was a bit of a threshold, now we’ve experienced the added value of it.”*

*106: “The chat function is really invented during the corona crisis. I know that sometime earlier we did talk about it but there was still… Well, it never really was in the planning. Then it was accelerated in the planning and got implemented quickly.”*

All professionals mentioned difficulties and disadvantages of using online tools / eHealth. They described how they think their clients mostly prefer face-to-face contact, how technical difficulties can undermine care and support processes, and how online contact isn’t suitable for everyone (for example older people, (digital) illiteracy, people who do not speak the language, and people with no access to a computer or smartphone). Also, as is stated in the previous section of this article, professionals can find it hard to use online tools and eHealth and some felt like online contact isn’t as effective as face-to-face contact.

*109: “If there’s really complex problems at hand, for example intimate partner violence which entails a systemic component as well, I think you cannot completely do that online. If for example it is about mild anxiety or something like that maybe you could perfectly do that completely online, with videocalls.”*

*107: “I hear professionals dislike that they cannot assess the family’s situation from a screen. With a home visit you can see whether it is clean, how does it smell? There are many more signs that you get from an one on one conversation that you do not see via eHealth. So I know from professionals I’ve spoken with that they do it out of necessity but they’d rather do home visits. Maybe it would work for part of the care.”*

There were also advantages of online contact according to all professionals. For example, it is easier to schedule appointments, clients are able to do more at home, and it lowers the threshold. Furthermore, one professional said some people were hesitant about using eHealth and thought they weren’t equipped for using it but now that they had no choice, they discovered their proficiency in using these means.

*101: “I like that people can do more from home and they are not as hesitant anymore. Now they have to and the threshold is lower, they’re more actively involved in their treatment. Also, there are less cancellations because scheduling appointments is easier and people have less reasons to miss their appointment, for example the car broke down. So I find it is an added value that I really talk with clients more often.”*

*109: “When it is difficult to seek help an online option can fill the gap and motivate someone to seek help. Or provide help, also when someone wants to stay anonymous. I think that’s the most important function of the online part.”*

*114: “I think for the most severe cases it wouldn’t be sufficient but it could provide much safety for a lot of people because it is online. That way they’re more likely to share their experience or seek help, as a helping hand. So I’m definitely in favor of it.”*

As added value of online tools, professionals named a lower threshold, the option to easily provide information (in multiple languages), anonymity, and easy access (also in times of a pandemic).

*106: “I think it is important that there are many ways available to seek help, digital ways are very important as well. That’s why we implemented a chat function. If you live with other people it is harder to pick up the phone, that’s also the case without corona. People experience pretty high thresholds for calling while a chat is easy.”*

*114: “You do not have to leave your house and with regard to corona, you’re not at risk of infection or infecting someone else. Maybe the distance could also make it safer to cross the threshold, like: I want to have a look at this app but on my terms and when it suits me and then I would want to tell something. It can be that needed push.”*

*110: “eHealth could mean a lot when it is available in multiple languages. They’re already in a difficult position, they do not know where to go, and if you have to explain everything in Dutch that’s not feasible. Those people should receive help in their mother tongue first because that’s what you go back to when you’re panicking.”*

However, all professionals stated that online tools and eHealth cannot and should not replace face-to-face contact, although some think this depends on personal preferences. They generally encouraged using online tools and eHealth as a stepping stone in help seeking and in the context of blended care (face-to-face combined with online), especially in the case of DVA. One professional thought online help can be especially helpful for vulnerable groups. Furthermore, another professional emphasized the need for critical evaluation of online tools and eHealth and the help options they present, as people in stressful situations such as DVA are not always able to do this themselves.

*112: “I think it helps either way if you can find information. I do feel that personal contact is necessary as well because with domestic violence it helps to not be alone in this. And if you’re alone on your computer and it stays inside, between you and your computer, I think it would help too little.”*

*115: “I think it can certainly be good and valuable. It depends on the situation. I can imagine that online help lowers the threshold to go to an organization. On the other hand, it could decrease someone’s motivation because online help is also more distant in a certain way. I think the combination could be good.”*

According to professionals, prerequisites and stimuli for getting people to use eHealth or online tools in the context of DVA or IPVA are present at multiple levels. For example in 1) awareness and accessibility: ability to raise awareness amongst the target group, a low threshold, and anonymity; 2) in accommodating to the survivors’ needs: a quiet environment, context sensitivity (for example language and cultural background), and knowledge about their privacy, safety and autonomy when using eHealth; and 3) in providing sufficient care: expertise, psycho-education, stories from other DVA survivors, help options, an emphatic component, and a professional you can contact. A few also mentioned that professionals stimulating the use of eHealth is important for a successful implementation.

*101: “We should offer it by default. If you take a look at it together there wouldn’t be a threshold to use it anymore. (…) I think people in that situation often feel unsafe so it is very important to discuss safety and privacy. It is important that we set the norm and provide eHealth as something that’s part of it and then people will use it.”*

*107: “I think it is also very important that an emphatic professional is present to lend an ear and to calm someone. A professional that could immediately take action when it is needed. Often you do not have the clarity of mind yourself to think about what you need now. So I think a human component, an emphatic component really is essential.”*

*115: “They’re a bit traumatized and they often cannot see the wood for the trees. I think it would be very good if structure is created in their search. So I would say: let them arrive at a help option that can support them in working out what they need and want.”*

## Discussion

The COVID-19 pandemic not only globally influenced the prevalence and severity of DVA, it also affected the professionals who work in the field of DVA. Research primarily focuses on survivors, while professionals who are responsible for detecting, reporting, registering, monitoring, and preventing DVA, and providing care, help and support are rarely asked about their experiences. However, this perspective is essential towards professionals’ occupational and mental health and to optimize care for DVA survivors, especially during a pandemic. We explored the perceptions of professionals of the survivors’ experience and the work-related issues.

### Domestic violence and abuse during the COVID-19 pandemic

The COVID-19 pandemic is a risk factor for an increase in DVA, due to increased stress, psychological problems, social isolation and financial problems, as well as reduced social support and access to help [11, 19, 37–39]. The World Health Organization (WHO) has reported a 60% increase in emergency calls from women subjected to IPVA in April 2020 [40]. Countries near the Netherlands (the UK, Denmark, Germany, Belgium and France) also reported proof or at least signs of a rise in DVA [41–47]. In the Netherlands however, no increase in DVA cases has been registered by *Veilig Thuis* (VT; translation: Safe At Home; national DVA organization) or the police until now [19–21], which is consistent with the views expressed by the professionals in this study. However, a lack of reporting does not prove the absence of a rise of DVA in the Netherlands during the lockdown. While some professionals think the pandemic might not have had a significant impact on DVA, most professionals do notice increased stress, more acute crisis situations, more excessive violence, a surge in the use of online DVA chats, and more phone calls to DVA organizations other than VT or the police.

Other sources also imply that while the total number of cases might not have risen, DVA has become more severe and there are indications for a non-reported DVA rise in the Netherlands. Several local (VT) organizations now describe an increase in more acute situations, and doctors in hospitals report more serious forms of child abuse [19, 48–51]. Nationwide, VT has recently seen a rise in phone calls and use of their newly opened chat function, although not all calls are by survivors themselves and sometimes unrelated to DVA [19, 27, 50, 52]. One of the professionals mentioned that some people started using alcohol and drugs earlier in the day. Research in Australia linked the increase of alcohol use during the pandemic to the increase of DVA [53]. Last, based on signs of DVA detected by childcare professionals, child abuse has increased significantly over the first lockdown. The majority of these cases entailed emotional abuse, including witnessing DVA [54]. In most families (57%) IPVA and direct child abuse occur together [55]. This implies a potential increase of IPVA that was not detected by the current options of the support system.

The current system for identifying, registering and reporting DVA might be suboptimal in reaching DVA survivors and bystanders, and therefore in its capability of providing complete figures of (the prevalence of) DVA. McLay [56] states that official DVA reports are incomplete: “the question here may be less about occurrence and more about reporting” of DVA while taking the source, for example police vs. hotline data, into account. A professional in this study says that we are too reliant on professionals for DVA reports. Most reports to VT indeed come from the police (66,5% in 2020) and professionals (24,4% in 2020) [57]. But professionals were less able to identify signs of DVA during the pandemic and might experience obstacles to report DVA and child abuse in general, for example because of a lack of expertise, the fear that it would damage the relationship with the patient / client / family, or thinking it is not their role to report [58–64]. Furthermore, professionals may not always trust VT and they do not seem to be very familiar with the option of (anonymously) calling VT for advice [65].

Besides, most survivors seek support from their informal support network, not primarily from the police or VT [28, 66], even in the case of a recent incident of physical or sexual violence [67]. Figures from Amsterdam in 2018 show that on average 33 incidents of DVA have occurred before a survivor goes to the police to press charges [68]. Furthermore, despite VT being the official reference for reporting DVA in the Netherlands, it could be the case that the general public isn’t very familiar with VT and what VT does yet, especially among people with a migrant background [69–74]. Thus, the official figures provided by the police and VT are probably an underrepresentation of the actual DVA situation in the Netherlands due to systemic barriers.

### Working conditions and the use of eHealth / online tools during the rise of the COVID-19 pandemic

Professionals’ working conditions have changed significantly due to the sudden switch to working from home and working online. They had to make an extra effort for acquainting themselves with new systems and implementing them, and to align these new practices with collaborative partners while respecting privacy and safety regulations. Not all of the organizations were prepared for this sudden switch. The diminished quality of the internet and phone connection and sometimes having to temporarily discontinue treatment was frustrating for both clients and professionals. Furthermore, some professionals find the amount of time spend looking at a screen very challenging. Working from home led to feelings of loneliness and posed a challenge in creating a healthy work-life balance. They worried about their clients’ wellbeing and some feared they could provide insufficient care or impaired service quality. They worried that they missed signs of DVA because of the distance. Furthermore, professionals working on location sometimes had to manage clients who were reluctant to respect the COVID-19 measures, leading to tension and possibly endangering the professionals and their relatives. All of this led to an increased (psychological) burden on the professionals working in the field of DVA, while having less opportunity to discuss these difficulties with colleagues / peers. These outcomes are very consistent with findings from studies carried out in the UK and the Netherlands among mental healthcare and DVA professionals [19, 75, 76]. One of the Dutch researchers from the Trimbos institute study [75] states that *“the complaints of mental healthcare professionals are similar to what intensive care professionals experience. … It is understandable that we focus on the professionals in physical healthcare but we also really need our mental healthcare professionals, now and in the future.”* [77]. Thus, it is clear that DVA and mental healthcare professionals are in a worrisome situation during this pandemic which is detrimental for both the professionals and their clients, especially since this crisis isn’t short-lived.

While suddenly working online much more than usual created challenges and online help is seen as an addition and not as a replacement, professionals see advantages and opportunities in using eHealth and online tools. For example, congruent with the professionals interviewed by the Verwey Jonker institute, some professionals found that communication and meetings with colleagues were more efficient and quicker online [19]. Professionals think that while online help isn’t for everyone, it could be helpful for vulnerable people, for less complex problems, in help seeking (lower threshold), in scheduling appointments, less no-shows, more autonomy for clients, and higher client involvement in treatment.

Perhaps professionals would feel more confident about their own skills and the benefits and effectiveness of using online tools and providing online help if they would receive adequate training [30]. Professionals say they’re interested in blended care: combining online and offline care. With regard to DVA, they feel that online interventions aimed at providing information, help options, and support can have a stepping stone function for DVA survivors in help seeking and could fit with a blended care approach. Their ideas on what is needed to successfully use eHealth in the context of DVA or IPVA is very similar to the outcomes of a Dutch qualitative study among IPVA survivors and professionals [78], which was used to inform the development of an eHealth intervention (SAFE). Developing sufficient eHealth interventions and adequately training professionals in providing online help might reduce their experienced stress and service-related worries. When professionals feel more competent about their online skills this also facilitates optimized use of eHealth and online tools with advantages for professionals and clients and creating more ways for DVA survivors to seek and receive help.

### Limitations

The fluctuating measures to restrict the COVID-19 pandemic create a situation where timing and local context significantly affect the interpretation of the outcomes of this study. The summer period in the Netherlands was accompanied by a lifting of many measures after the first lockdown. However, the second lockdown that is still in effect in March 2021, creates a different context and the answers of some of the professionals might change as the pandemic and its containment measures progress. Findings about a possible increase of DVA and child abuse in the Netherlands during the COVID-19 pandemic are still accumulating and little information was available to the professionals at the time of the interviews. Furthermore, a limited number of professionals was interviewed, which is a limitation for generalizability, but it is varied in professions and provides perspectives from different contexts. Furthermore, no data was gathered on DVA perpetrators’ help seeking behavior and professionals’ experiences in this regard and it could be helpful to investigate this and assess whether perpetrators seeking help to stop the violence have sufficient access to professional help and support during the pandemic.

### Implications

#### Reporting DVA

For a more complete image of DVA in the Netherlands and to better support DVA survivors a critical evaluation of the current report system is needed. Interestingly, the Dutch 2020 Impact Monitor Domestic Violence and Child Abuse stresses that *“… the reach of Veilig Thuis as a hotline for advice and reporting should be as big as possible; everyone with a suspicion of domestic violence or child abuse must find the way to Veilig Thuis as soon as possible.”* (p. 18; [28]);, which places the duty of reporting on bystanders and professionals and does not appropriately target survivors themselves.

The recent Regioplan report focused on concretizing the recommendations in the earlier report from the Group of Experts on Action against Violence against Women and Domestic Violence [79], states that research into the official report system and possible obstacles for survivors in reporting and help seeking is needed. Furthermore, they advise to take away learning points from DVA hotlines abroad with a low threshold, for example in Sweden, where the majority of callers are survivors and more than half of the women in the general population know about this hotline [80]. In 2019 in the Netherlands, the SAFE eHealth intervention became available for women who experience IPVA, containing information, experiences, vlogs, situational checklists and help options [32]. For survivors, a low threshold and a trustworthy institution is a precondition to contact a hotline or visit a website. For example, by guaranteeing anonymity, a primary focus on safety and wellbeing, control over the process and the interventions initiated, and the protection of their children. For now, it seems the current system is primarily focused on reports from professionals and lacks the ability to connect with survivors.

#### Supporting professionals

To support professionals in their changed working conditions it is important that working from home and working online is optimally facilitated. For example, by helping them to create a private and sufficiently equipped workplace; by creating options to easily meet colleagues (online) for discussion and peer group coaching, and by providing confidential counselling or psychological help when needed.

The need to work on-site as an essential worker during a pandemic can significantly increase work-related stress [81, 82], especially when clients are not willing to adhere to the COVID-19 rules when they visit a professional. Thus, it is important that employers make an effort to provide a safe work environment at the workplace.

Working online and using eHealth requires sufficient technical support and safe systems for online contact. Furthermore, sufficiently training professionals, in both basics of using online tools as well as in providing actual online help, will improve the quality and effectiveness of online help and it can motivate professionals and increase their confidence in providing this type of help.

## Conclusions

During the COVID-19 pandemic, it is likely that DVA has increased both in numbers and severity, although official reports in the Netherlands did not show this. The use of eHealth and online tools has increased dramatically, expanding the knowledge in this field and enhancing the quality of online help and support, which is beneficial for a low threshold type of care. More research on eHealth in the context of DVA remains necessary to assess its effectiveness and challenges for clients and professionals. In improving the support for DVA survivors we should critically evaluate our reporting system and its accessibility for survivors, improve options for professionals to detect DVA in times of social distancing, and we should further explore eHealth and online help directed at survivors. Finally, since we greatly rely on DVA professionals and professionals from related fields to identify and report DVA and to provide the much-needed help and support, even in challenging circumstances, we owe it to them to optimize their working conditions.

## Supplementary Information

**Additional file 1 **: **Supplementary material**. Interview guide (main questions – translated to English). Interview guide used for the semi-structured interviews, main questions (originally in Dutch) are translated to English.

## Data Availability

The English translation of the interview guide with the main questions is available and is added as supplementary material (Additional file 1). The data that support the findings of this study consist of audio files and transcripts of the interviews conducted and are available on request from the corresponding author NvG. The data are not publicly available due to them containing information that could compromise research participant privacy or consent.

## Abbreviations

DVA: Domestic violence and abuse
GP: General practitioner
IPVA: Intimate partner violence and abuse
GREVIO: Group of Experts on Action against Violence against Women and Domestic Violence
PPE: Personal protective equipment
UK: The United Kingdom
US: The United States
VT: *Veilig Thuis* (English translation: Home Safe)
WHO: World Health Organization
