# Supplementary material for: Professionals’ views on working in the field of domestic violence and abuse during the first wave of COVID-19: a qualitative study in the Netherlands
Source: BMC Health Serv Res. 2021 Jun 30;21:624. doi: 10.1186/s12913-021-06674-z (PMC8241882; doi:10.1186/s12913-021-06674-z)
Supplement: Supplementary file 1 — Additional file 1 : Supplementary material. Interview guide (main questions – translated to English). Interview guide used for the semi-structured interviews, main questions (originally in Dutch) are translated to English. [file 12913_2021_6674_MOESM1_ESM.pdf]

Additional file 1.

Supplementary material: interview guide (main questions – translated to English).

The interviewer introduced herself and the project that the interview study is part of.

1. Can you introduce yourself? (age, organization, region of work, position)
2. Can you tell me something about the organization you're working for?
3. Has your workday changed since the pandemic?
   - If so, can you tell me what has changed?
   - If not, are there tasks that you cannot perform during the pandemic?
4. Do you think new problems have arisen because of these changes?
5. Do you feel like you were able to provide sufficient care and support with these changes?
6. Do you / does your organization currently see an increase in domestic violence and abuse?
   - If so, how big do you think this increase is and what do you think the cause is of this increase?
   - If not, do you expect an increase at a later point in time?
7. Do you worry about specific groups during this pandemic?
8. Are you familiar with eHealth?
9. What is your opinion on eHealth in the context of domestic violence and abuse?
10. Does your organization use eHealth and what are your experiences with it?
11. Do you think eHealth is a good (additional) option during the pandemic?
    - Why (not)?
12. What do you think is needed to encourage people to use eHealth?
13. What do you think is needed to support women via eHealth?
14. Are you familiar with the SAFE eHealth intervention?

    - Do you think it could complement regular care and support? Why (not)?

15. Do you have any last remarks or things we didn’t ask about but that you think are important to discuss?
